# Quinio

**Published:** 1860-05

**Authors:** 


*Quinio.*—Tshudi, who has just returned from an exploring expedition in South America, exhibited to the Vienna Academy a preparation of an Italian chemist at La Paz, of which the following is the history:

*Quinio* is a substance obtained from the *fresh* barks of Bolivian Cinchonæ simply by exhaustion with lime and alcohol, and contains quinia in so large a proportion that it is only required to boil it with dilute sulphuric acid in order to obtain sulphate of quinine, pure and in very handsome crystals.

It is a resin-like substance of a yellow color and the lustre of wax, of a bitter taste, which, on account of its resinous, insoluble nature, is long retained on the palate. It is almost insoluble in cold, and but little more in boiling water, to which it imparts a bitter taste, but completely soluble in alcohol, and also in ether, from which latter solution it is, however, partly precipitated again with a yellow color when exposed to the rays of the sun. From its solution in alcohol it is precipitated by water as a mllk. In dilute sulphuric acid it dissolves with a yellow color, leaving behind but a few flakes of a waxy-ethereal odor. The dilute solution in sulphuric acid gives a white precipitated carbonate of soda which agglutinates and is then of a dirty white color, solu ble in ether, which latter solution yields, on evaporation, quinia in a state of perfect purity.

Quinio is free from cellulose, differing in this from the bark and the chinoidine of commerce. From a chemical analysis and its behavior with various reagents, it appears to be identical with Liebig's pure chinoidine, the latter being far more impure.—*Chemisches Centralblatt, Dec. 7th*, 1859.

